# A soluble truncated tau species related to cognitive dysfunction and caspase-2 is elevated in the brain of Huntington’s disease patients

**DOI:** 10.1186/s40478-019-0764-9

**Published:** 2019-07-30

**Authors:** Peng Liu, Benjamin R. Smith, Eric S. Huang, Abhishek Mahesh, Jean Paul G. Vonsattel, Ashley J. Petersen, Rocio Gomez-Pastor, Karen H. Ashe

**Affiliations:** 1 Departments of Neurology, University of Minnesota, Minneapolis, MN 55455 USA; 2 Departments of Neuroscience, University of Minnesota, Minneapolis, MN 55455 USA; 3 N. Bud Grossman Center for Memory Research and Care, University of Minnesota, Minneapolis, MN 55455 USA; 4 Division of Biostatistics, University of Minnesota, Minneapolis, MN 55455 USA; 5 Wayzata High School, Plymouth, MN 55446 USA; 6 Department of Pathology, Presbyterian Hospital and Columbia University, New York City, NY 10032 USA; 7 Geriatric Research, Education, and Clinical Centers, Veterans Affairs Medical Center, Minneapolis, MN 55417 USA; 8 Current address: University of Miami, Miller School of Medicine, Miami, FL 33136 USA

**Keywords:** Microtubule-associated protein tau, Caspase-2, Huntingtin, Frontal cortex, Caudate nucleus, Striatum, Huntington’s disease, Immunoprecipitation, Western blotting, Truncation

## Abstract

Huntington’s disease (HD) is a progressive neurodegenerative disease. Involuntary movements, cognitive impairment and psychiatric disturbance are the major clinical manifestations, and gradual atrophy and selective neuronal loss in the striatum and cerebral cortex are the pathologic hallmarks. HD is caused by expanded CAG trinucleotide repeats at the N-terminus of *IT15* that encodes the huntingtin (HTT) protein, though the molecular mechanisms through which the mutant HTT (mHTT) exerts toxic effects remain obscure. Members of the caspase family, including caspase-2 (Casp2), play an important role in HD pathogenesis. Genetic ablation of Casp2 ameliorates cognitive and motor deficits of HD mice, though the molecular targets of Casp2 are still unclear. It is well established that the microtubule-associated protein tau potentiates cognitive dysfunction in a variety of neurodegenerative disorders, including HD. Our recent study indicates that Casp2-catalyzed tau cleavage at aspartate 314 (tau 2N4R isoform numbering system) mediates synaptotoxicity, cognitive deficits and neurodegeneration in cellular and mouse models of frontotemporal dementia; further, levels of Δtau314, the soluble, N-terminal cleavage product, are elevated in individuals with mild cognitive impairment and Alzheimer’s disease, compared with cognitively normal individuals. Here, we identified the presence of Δtau314 proteins in the striatum (caudate nucleus) and prefrontal cortex (Brodmann’s area 8/9) of human subjects, and showed that in both structures, levels of Casp2 and Δtau314 proteins correlate well, and both proteins are higher in HD patients than non-HD individuals. Our findings advance our understanding of the contribution of Casp2-mediated Δtau314 production to HD pathogenesis.

**Electronic supplementary material:**

The online version of this article (10.1186/s40478-019-0764-9) contains supplementary material, which is available to authorized users.

## Introduction

Huntington’s disease (HD) is an autosomal-dominant and progressive brain disorder. The clinical manifestations of HD involve uncontrolled movements, psychiatric disturbance and cognitive decline (for review, see for example [39]); and HD is pathologically characterized by the gradual atrophy of and selective neuronal loss in the neostriatum (caudate nucleus and putamen) and cerebral cortex (for review, see for example [33]). HD is caused by an abnormal expansion of CAG trinucleotide repeats at the N-terminus of *IT15* (*interesting transcript 15*) encoding the huntingtin (HTT) protein [15]. However, the molecular mechanisms through which the mutant HTT (mHTT) leads to the onset and development of HD are not fully understood (for review, see for example [18]).

Cysteine-dependent aspartate-directed proteases (caspases) play an important role in HD pathogenesis. Hydrolysis of mHTT by caspases-2, − 3 and − 6 [41, 42] promotes the aggregation of its N-terminal cleavage products in the brain [10, 12, 14, 19]. Interestingly, while caspase-2 (Casp2) cleaves mHTT, blockage of Casp2-mediated mHTT cleavage did not affect disease progression [12]. Yet, genetic ablation of Casp2 led to amelioration of cognitive and motor dysfunctions of transgenic mice overexpressing *IT15* with expanded CAG trinucleotides [8]. Thus, it appears that Casp2 mediates the behavioral abnormalities. However, the substrate, whose cleavage by Casp2 leads to the abnormalities, is unknown.

The microtubule-associated protein tau (MAPT) is a highly soluble, unstructured molecule that is primarily present in the neurons of the central nervous system. The contribution of tau to HD pathogenesis involves, but is not limited to, the presence of aggregated tau inclusions in the brain of HD patients [7, 9, 11, 17, 23, 25, 26, 31, 38], the influence of *MAPT* haplotype in cognition of HD patients (patients with the H2 haplotype are more severely cognitively impaired than those with the H1 haplotype) [38], the amelioration of behavioral abnormalities of HD mice by genetic ablation of *Mapt* [11], and the mHTT-mediated alterations in tau splicing isoform expression [11] and tau hyperphosphorylation [6, 13]. Despite these findings, the exact tau species and the molecular mechanisms through which tau mediates HD pathogenesis are still largely unknown. The association of tau with cognitive dysfunction has been well established in a variety of mouse lines modeling multiple neurodegenerative diseases, and tau proteolysis and acetylation were shown to mediate cognitive impairment of animals [24, 32, 35, 43, 44].

Of particular note, Casp2-mediated tau cleavage at amino acid residue aspartate 314 (D314) (tau 2N4R isoform numbering system hereafter unless specified) is responsible for synaptic function impairment and cognitive deficits in cellular and transgenic mouse models of frontotemporal dementia and parkinsonism linked to chromosome 17 [44], indicating a toxic partnership between Casp2 and tau [36]. In addition, Δtau314 proteins, the soluble cleavage products that are immunoreactive to monoclonal tau-13 (a.a. 15–25), were shown to be related to Alzheimer’s disease (AD), as the levels of Δtau314 proteins are elevated in the disease-affected inferior temporal gyrus region of individuals with mild cognitive impairment (MCI) and AD patients compared to cognitively normal, elderly individuals [44].

In this study, we identified the presence of Δtau314 proteins in two HD-affected brain regions (caudate nucleus and prefrontal cortex (Brodmann’s area (BA) 8/9)) of human subjects using conventional immunoprecipitation (IP)/Western blotting (WB) analysis. We showed that HD patients have higher levels of Δtau314 proteins as well as Casp2 than non-HD individuals. We also showed that levels of Casp2 and Δtau314 proteins are highly correlated. Results of this study, together with previous findings, suggest that Casp2-mediated tau cleavage to produce Δtau314 is a common pathologic mechanism that leads to synaptotoxicity in multiple neurodegenerative diseases.

## Materials and methods

### In vitro synthesis of human full-length tau and Δtau314 proteins

Complementary DNA (cDNA) encoding the full-length tau 0N4R isoform was cloned into the pcDNA3.1(+) mammalian expression vector (Thermo Fisher Scientific, Waltham, MA). For synthesis of the truncated human tau protein Δtau314, the trinucleotides encoding amino acid residue leucine 315 were modified to a stop codon using the QuikChange II Site-Directed Mutagenesis Kit (Agilent Technologies, Santa Clara, CA) following the manufacturer’s instructions. The following primers were used for the mutagenesis: forward primer, 5′ - AGTCTACAAACCAGTTGACTAGAGCAAGGTGACCTCCAAG - 3′; reverse primer, 5′ - CTTGGAGGTCACCTTGCTCTAGTCAACTGGTTTGTAGACT - 3′. Protein synthesis was performed using the TnT Coupled Reticulocyte Lysate Systems (Promega, Madison, WI) following the manufacturer’s instructions.

### Human brain collection

For the small cohort pilot study, frozen post-mortem specimens of dorsolateral prefrontal cortex (BA 8) from ten de-identified subjects—five HD patients and five non-HD individuals—were obtained from the Institute of Neurobiology Brain Bank (HUB-ICO-IDIBELL Biobank), Bellvitge University Hospital, Hospital del Llobregat, Spain.

For the large cohort study, frozen post-mortem specimens were obtained from the National Institutes of Health (NIH) NeuroBioBank and the New York Brain Bank (NYBB) at Columbia University, New York City, New York. Dorsolateral prefrontal cortex (BA 8) and caudate nucleus tissue from twenty-four de-identified subjects were provided by the NIH NeuroBioBank, and dorsolateral and medial prefrontal cortex (BA 9) tissue from twenty de-identified subjects were provided by NYBB. Tissue specimens from thirteen HD patients and fourteen non-HD individuals (see Additional file 1: Table S1 for detailed demographic characteristics) were used in the study based on the following criteria: 1) the age at death, sex distribution and post-mortem interval (PMI) of brain tissue harvest of the HD patients are not significantly different from those of the non-HD individuals; 2) The ages of subjects are 50 years or older, and 3) the PMIs of brain tissue harvest are less than 22 h.

Frozen tissue was stored at − 80 °C in either polypropylene bags or cryotubes prior to shipment to the University of Minnesota, Twin Cities, Minnesota.

All procedures were approved by the Institutional Review Boards (IRBs) of the HUB-ICO-IDIBELL Biobank (Spain), the Columbia University, the University of Miami, the Department of Veterans Affairs – Los Angeles and the University of Minnesota.

### Brain protein extraction

Aqueous brain protein extracts were prepared based on a protocol previously published [34]. Specifically, human tissue specimens of 0.3–0.5 g were cut (or hemi-forebrains of rTg4510 mice were dissected), transferred to 5 volumes (1 g per 5 mL) of ice-cold extraction buffer (25 mM tris(hydroxymethyl)aminomethane-hydrochloric acid (Tris-HCl), pH 7.4; 140 mM NaCl and 3 mM KCl with the following protease and phosphatase inhibitors: 0.1 mM phenylmethylsulfonyl fluoride, 0.2 mM 1,10-phenanthroline monohydrate, a protease inhibitor cocktail (MilliporeSigma, Burlington, MA) and two phosphatase inhibitor cocktails (MilliporeSigma)), and homogenized using a Dounce homogenizer. The resulting material was centrifuged for 90 min (16,100 *g*, 4 °C). The supernatant was subsequently depleted of endogenous immunoglobulin G (IgG) with Protein G Sepharose 4 Fast Flow beads (GE Healthcare, Piscataway, NJ; 50 μL of slurry per 500 μL of sample), and stored at − 20 °C. Protein concentrations of brain extracts were determined using a bicinchoninic acid protein assay kit (Thermo Scientific, Rockford, IL) according to the manufacturer’s instructions.

### Immunoprecipitation

To detect and measure levels of Δtau314 and soluble tau proteins, 173 μg of proteins from the aqueous brain extracts were brought to a volume of 500 μL through the addition of immunoprecipitation (IP) dilution buffer (50 mM Tris-HCl, pH 7.4; 150 mM NaCl) containing the protease and phosphatase inhibitors listed in Section *Brain protein extraction*. The resulting samples were incubated with 10 μg of monoclonal antibody tau-13 (BioLegend, San Diego, CA) or mouse IgG (negative control). Immuno-complexes were captured by incubation with 30 μL of Protein G Sepharose 4 Fast Flow resin at 4 °C for 14–16 h. Following capture of immuno-complexes, resins were gently washed sequentially in IP buffer A (50 mM Tris-HCl, pH 7.4; 300 mM NaCl; 1 mM ethylenediaminetetraacetic acid (EDTA) and 0.1% volume/volume (v/v) polyethylene glycol p-(1,1,3,3-tetramethylbutyl)-phenyl ether (Triton X-100) with protease and phosphatase inhibitors) and IP buffer B (50 mM Tris-HCl, pH 7.4; 150 mM NaCl; 1 mM EDTA and 0.1% (v/v) Triton X-100 with protease and phosphatase inhibitors) at 4 °C for 5 min. Complexes were then eluted by boiling the magnetic beads at 95 °C in 30 μL of sodium dodecyl sulfate (SDS)-polyacrylamide gel electrophoresis (PAGE) loading buffer (500 mM Tris-HCl, pH 8.0; 24% (v/v) glycerol; 8% weight/volume (w/v) SDS; 0.01% (w/v) Coomassie brilliant blue; 0.1% (v/v) phenol red; 710 mM β-mercaptoethanol); elutates were size-fractionated on 10% Criterion Tris-HCl Precast gels (Bio-Rad, Hercules, CA), and electrophoretically transferred onto 0.2-μm nitrocellulose membranes at a constant current of 0.4 A for 4 h at 4 °C.

### Western blotting

Western blotting (WB) was performed according to a previously published protocol [21].

To measure levels of Δtau314 and soluble total tau (T-tau) proteins, blots were probed with biotin-conjugated 4F3 (1:45,000, final concentration = 36 ng/mL) and biotin-conjugated tau-5 (Thermo Fisher Scientific; 1:30,000, final concentration = 17 ng/mL), respectively. Following 5 X 5-min washes in wash buffer (10 mM Tris-HCl, pH 7.4; 200 mM NaCl; 0.1% (v/v) polyoxyethylene (20) sorbitan monolaurate (Tween 20)), membranes were incubated at room temperature in horseradish peroxidase (HRP)-conjugated NeutrAvidin (Thermo Fisher Scientific; 1:5,000, final concentration = 0.2 μg/mL) for 10 min. The membranes were then washed again (5 X 5-min in wash buffer) prior to being developed.

To measure levels of Casp2, 100 μg of proteins from the aqueous brain extracts were electrophoretically separated by SDS-PAGE using Criterion 10% Tris-HCl Precast gels (Bio-Rad), and then transferred onto nitrocellulose membranes. Blots were probed with rabbit monoclonal anti-Casp2 (Abcam, Cambridge, MA; 1: 5,000, final concentration = 496 ng/mL). Following 5 X 5-min washes in wash buffer, membranes were incubated at room temperature in HRP-conjugated anti-rabbit IgG (Thermo Fisher Scientific; 1:200,000, final concentration = 5 ng/mL) for 1 h. The membranes were then washed again (5 X 5-min in wash buffer) prior to being developed.

To measure levels of glyceraldehyde 3-phosphate dehydrogenase (GAPDH), 20 μg of proteins from the aqueous brain extracts were electrophoretically separated by SDS-PAGE using Criterion 10% Tris-HCl Precast gels (Bio-Rad), and then transferred onto nitrocellulose membranes. Blots were probed with rabbit monoclonal anti-GAPDH (Cell signaling Technology, Danvers, MA; 1: 4,000, final concentration = 25 ng/mL). Following 5 X 5-min washes in wash buffer, membranes were incubated at room temperature in HRP-conjugated anti-rabbit IgG (Thermo Fisher Scientific; 1:200,000, final concentration = 5 ng/mL) for 1 h. The membranes were then washed again (5 X 5-min in wash buffer) prior to being developed.

Western blots were developed using the West Pico electrochemiluminescence detection system (Thermo Fisher Scientific) except for the blots probed with biotin-conjugated 4F3 and anti-Casp2, which were developed using the West Femto detection system (Thermo Fisher Scientific). Signal intensities were quantified densitometrically using Optiquant (Packard Cyclone, Perkin-Elmer Life Sciences Inc., Boston, MA).

Experimenters performing IP/WB were blind to the demographic and clinical characteristics of subjects. The levels of proteins were determined from two independent experiments, and the mean levels were used for statistical analysis.

### Statistical analysis

Statistical analyses were performed using GraphPad Prism Version 7.04 (GraphPad Software, La Jolla, CA) and R Version 3.4.1 (R Foundation for Statistical Computing, Vienna, Austria). The demographic and neuropathologic characteristics were compared between HD patients and non-HD individuals using two-tailed, unpaired Mann-Whitney tests for continuous variables and two-sided Fisher’s exact tests for binary variables. The associations between demographic characteristics and levels of Δtau314 proteins (normalized to levels of T-tau proteins) were estimated using Spearman’s rank-order correlations for continuous variables and Mann-Whitney tests for binary variables. Finally, levels of Δtau314, T-tau, Casp2 and GAPDH were compared between HD patients and non-HD individuals using two-tailed, unpaired Mann-Whitney tests. Additionally, multiple linear regressions of the log transformed levels were fit with adjustment for the potential confounders of age at death, sex and PMI. The unadjusted version of this analysis—a two-tailed, unpaired *t*-test performed on the log transformed levels—is also presented. *P* values of less than 0.05 were considered statistically significant.

## Results

### Levels of Δtau314 proteins are elevated in the prefrontal cortex of HD patients in a small cohort

First, using a polymerase chain reaction (PCR)-based method previously published [40], we verified that the studied HD patients harbored > 35 CAG trinucleotide repeats in at least one allele, whereas the number of CAG repeats in the non-HD individuals did not exceed 35 in either allele (data not shown).

We then asked whether Δtau314 proteins were present in the prefrontal cortex of studied subjects. For this, we used 4F3 (Table 1), a monoclonal antibody that specifically targets truncated tau proteins ending C-terminally at D314, to detect Δtau314 proteins in the prefrontal cortex (BA 8) of five HD patients and five non-HD individuals, a cohort of the HUB-ICO-IDIBELL Biobank, Spain (Additional file 1: Table S1). Age at death, sex and PMI of brain tissue harvest in the HD patients and the non-HD individuals were matched (Additional file 1: Table S2).

**Table 1:** Antibodies used in this study

Antibody	Host/Isotype	Epitope^a^	Source
tau-13	Ms, IgG_1, κ_	Tau_15–25_	BioLegend, Cat. #: 835201
tau-5 (biotin-conjugated)	Ms, IgG_1_	Tau_210–241_	Thermo Fisher Scientific, Cat. #: MA5–12805
4F3 (biotin-conjugated)	Ms, IgG_2b, κ_	Tau_x-314_	Ashe laboratory
Anti-Casp2	Rb, IgG	C-terminus	Abcam, Cat. #: 179519
Anti-GAPDH	Rb, IgG	C-terminus	Cell Signaling Technology, Cat. #: 2118

^a^Amino acid residues of tau protein are counted using the 2N4R isoform numbering system

**Fig. 1:**
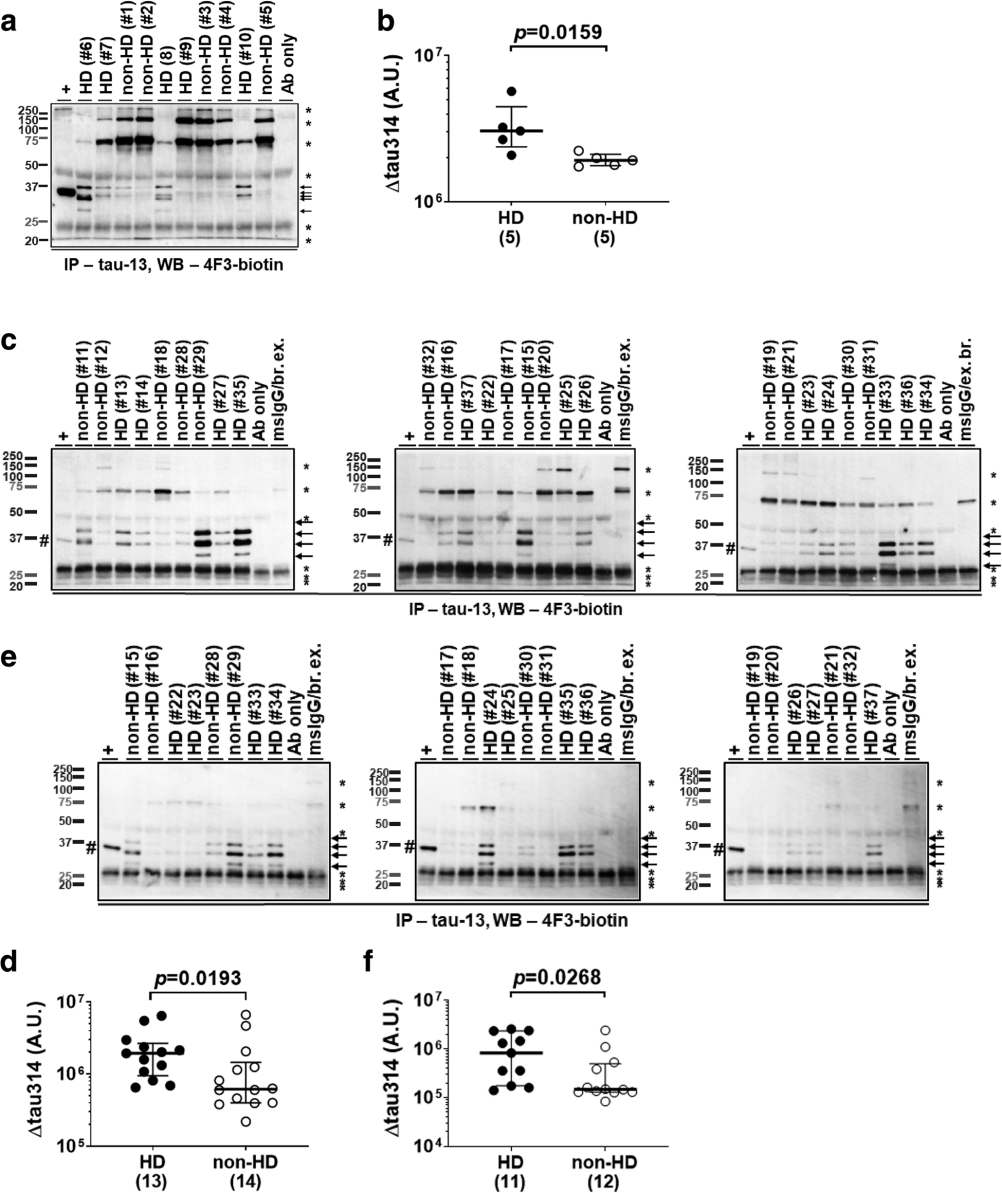
Levels of Δtau314 proteins are elevated in HD patients. **a**, **c**, **e** Representative IP/WB showing that the tau-13-immunoprecipitated Δtau314 proteins (arrows) were detected by the biotin-conjugated 4F3 antibody in the prefrontal cortex (Brodmann’s area (BA) 8) of subjects from the HUB-ICO-IDIBELL Biobank, Spain (**a**), in the prefrontal cortex (BA 8/9) of subjects from the NIH NeuroBioBank and the New York Brain Bank (**c**), and in the caudate nucleus of subjects from the NIH NeuroBioBank (**e**). HD, Huntington’s disease patients; non-HD, individuals without Huntington’s disease. +, in vitro synthetic Δtau314 proteins (hash; 20 μL of sample used in IP, positive control). *, non-specific bands. Sample IDs and disease diagnoses (Additional file 1: Table S1) were shown for IP reactions. Ab only, IP reaction containing only 10 μg of tau-13 (negative control). MsIgG/br. ex., IP reaction containing 10 μg of mouse IgG and 173 μg of soluble brain proteins (negative control). For the MsIgG/br. ex. lanes in figure c, brain extracts were a mixture of the nine samples studied in the same blot with each contributing 1/9th the amount (i.e., 19.2 μg); for the MsIgG/br. ex. lanes in figure e, 1/8th the amount (i.e., 21.6 μg). **b**, **d**, **f** Comparison of levels of Δtau314 proteins in the prefrontal cortex (BA 8) of HD patients and non-HD individuals from the HUB-ICO-IDIBELL Biobank, Spain (**b**), in the prefrontal cortex (BA 8/9), from the NIH NeuroBioBank and the New York Brain Bank (**d**), and in the caudate nucleus, from the NIH NeuroBioBank (**f**). The numbers of analyzed subjects are shown in parentheses. A.U. = arbitrary unit. The y-axes in figures are in log scale. Mann-Whitney test was used for between-group comparison; medians (middle long bars), and 1st (lower short bars) and 3rd (upper short bars) quartiles are shown

We identified 4F3-reactive Δtau314 proteins in all the studied subjects (Fig. 1a). The levels of Δtau314 proteins were elevated 1.6-fold in the HD patients compared to the non-HD individuals (Fig. 1b, Additional file 1: Table S3), and trended higher following adjustments for age at death, sex and PMI of brain tissue harvest (Additional file 1: Table S3).

### Levels of Δtau314 proteins are elevated in the prefrontal cortex of HD patients in a large cohort

We then attempted to confirm these findings in a larger cohort of subjects. Based on the quantitative analysis of 4F3-reactive Δtau314 proteins, we determined that the lowest sample size required for each group to achieve statistical significance under type I error α = 0.05 and power (i.e., (1-β)) = 0.80 is *N* = 11.

We measured levels of Δtau314 proteins in the prefrontal cortex (BA 8/9) of thirteen HD patients and fourteen non-HD individuals (Additional file 1: Table S1). Age at death, sex distribution and PMI of brain tissue harvest did not significantly differ between the HD patients and the non-HD individuals (Table 2). We found that levels of Δtau314 proteins were 3.1-fold higher in the HD patients than in the non-HD individuals (Fig. 1c and d, Table 3).

**Table 2:** Comparison of demographic characteristics of HD patients and non-HD individuals from the NIH NeuroBioBank and the New York Brain Bank used in the study of proteins in the prefrontal cortex (BA8/9)

	non-HD	HD	*p* value
Sample size, *N*	14	13	
Age at death [yr]:			
median (1st quartile, 3rd quartile)	71.5 (61.5, 79.5)	65.0 (60.0, 72.0)	0.12^a^
range	57.0–89.0	50.0–77.0	
Sex, female/male, No. (% female)	8/6 (47.1%)	9/4 (52.9%)	0.69^b^
Post-mortem interval [hr]:			
median (1st quartile, 3rd quartile)	12.6 (11.2, 18.1)	12.0 (9.2, 17.2)	0.68^a^
range	9.0–20.5	6.0–21.7	

^a^Two-tailed, unpaired Mann-Whitney test

^b^Two-sided Fisher’s exact test

**Table 3:** A statistical comparison of protein levels in the prefrontal cortex (BA8/9) of HD patients and non-HD individuals from the NIH NeuroBioBank and the New York Brain Bank

	Mann-Whitney^a^	*t*-test^b^	Multiple linear regression^c^
Δtau314	*p* = 0.019	*p* = 0.035	*p* = 0.094
T-tau	*p* = 0.011	*p* = 0.075	*p* = 0.099
Δtau314:T-tau	*p* = 0.00082	*p* = 0.013	*p* = 0.025
GAPDH	*p* = 0.46	*p* = 0.88	*p* = 0.35
Casp2:GAPDH	*p* = 0.0078	*p* = 0.0030	*p* = 0.019

^a^The two-tailed, unpaired Mann-Whitney test was performed

^b^The two-tailed, unpaired *t*-test with Welch’s correction was performed on the log transformed outcomes

^c^Multiple linear regression was used to analyze the log transformed outcomes of protein levels with adjustment for age at death, sex and PMI of brain tissue harvest

### Levels of Δtau314 proteins are elevated in the caudate nucleus of HD patients in a large cohort

Next, we measured levels of 4F3-reactive Δtau314 proteins in the caudate nucleus, a severely affected region, in a subset of the large cohort – eleven HD patients and twelve non-HD individuals (Additional file 1: Table S1). Age at death, but not sex distribution or PMI of brain tissue harvest, was significantly different between the HD patients and the non-HD individuals (Table 4). We found that levels of Δtau314 proteins in the caudate were 5.6-fold higher in HD patients than in non-HD individuals (Fig. 1e and f, Table 5).

**Table 4:** Comparison of demographic characteristics of HD patients and non-HD individuals from the NIH NeuroBioBank used in the study of proteins in caudate nucleus

	non-HD	HD	*p* value
Sample size, *N*	12	11	
Age at death [yr]:			
median (1st quartile, 3rd quartile)	74.0 (68.0, 80.5)	65.0 (58.0, 73.0)	0.04^a^
range	60.0–89.0	50.0–77.0	
Sex, female/male, No. (% female)	7/5 (58.3%)	7/4 (63.6%)	> 0.99^b^
Post-mortem interval [hr]:			
median (1st quartile, 3rd quartile)	13.8 (11.1, 18.6)	12.0 (9.2, 15.7)	0.44^a^
range	9.0–20.5	8.6–21.7	

^a^Two-tailed, unpaired Mann-Whitney test

^b^Two-sided Fisher’s exact test

**Table 5:** A statistical comparison of protein levels in the caudate nucleus of HD patients and non-HD individuals from the NIH NeuroBioBank

	Mann-Whitney^a^	*t*-test^b^	Multiple linear regression^c^
Δtau314	*p* = 0.027	*p* = 0.041	*p* = 0.082
T-tau	*p* = 0.00055	*p* = 0.0031	*p* = 0.038
Δtau314:T-tau	*p* = 0.0013	*p* = 0.00064	*p* = 0.0041
GAPDH	*p* = 0.15	*p* = 0.29	*p* = 0.29
Casp2:GAPDH	*p* = 0.0036	*p* = 0.0035	*p* = 0.0070

^a^The two-tailed, unpaired Mann-Whitney test was performed

^b^The two-tailed, unpaired *t*-test with Welch’s correction was performed on the log transformed outcomes

^c^Multiple linear regression was used to analyze the log transformed outcomes of protein levels with adjustment for age at death, sex and PMI of brain tissue harvest

### Levels of soluble total tau proteins are lower in the prefrontal cortex and caudate nucleus of HD patients in the large cohort

To ascertain whether the increases in Δtau314 proteins was due to an increase in soluble total tau (T-tau) proteins, we measured the levels of T-tau proteins that were immunoprecipitated by tau-13 antibody, and detected by tau-5, a monoclonal antibody directed against a mid-regional epitope of tau (Table 1). We quantified levels of T-tau proteins in the prefrontal cortex and caudate nucleus of subjects in the large cohort, and in the prefrontal cortex of subjects in the small cohort.

**Fig. 2:**
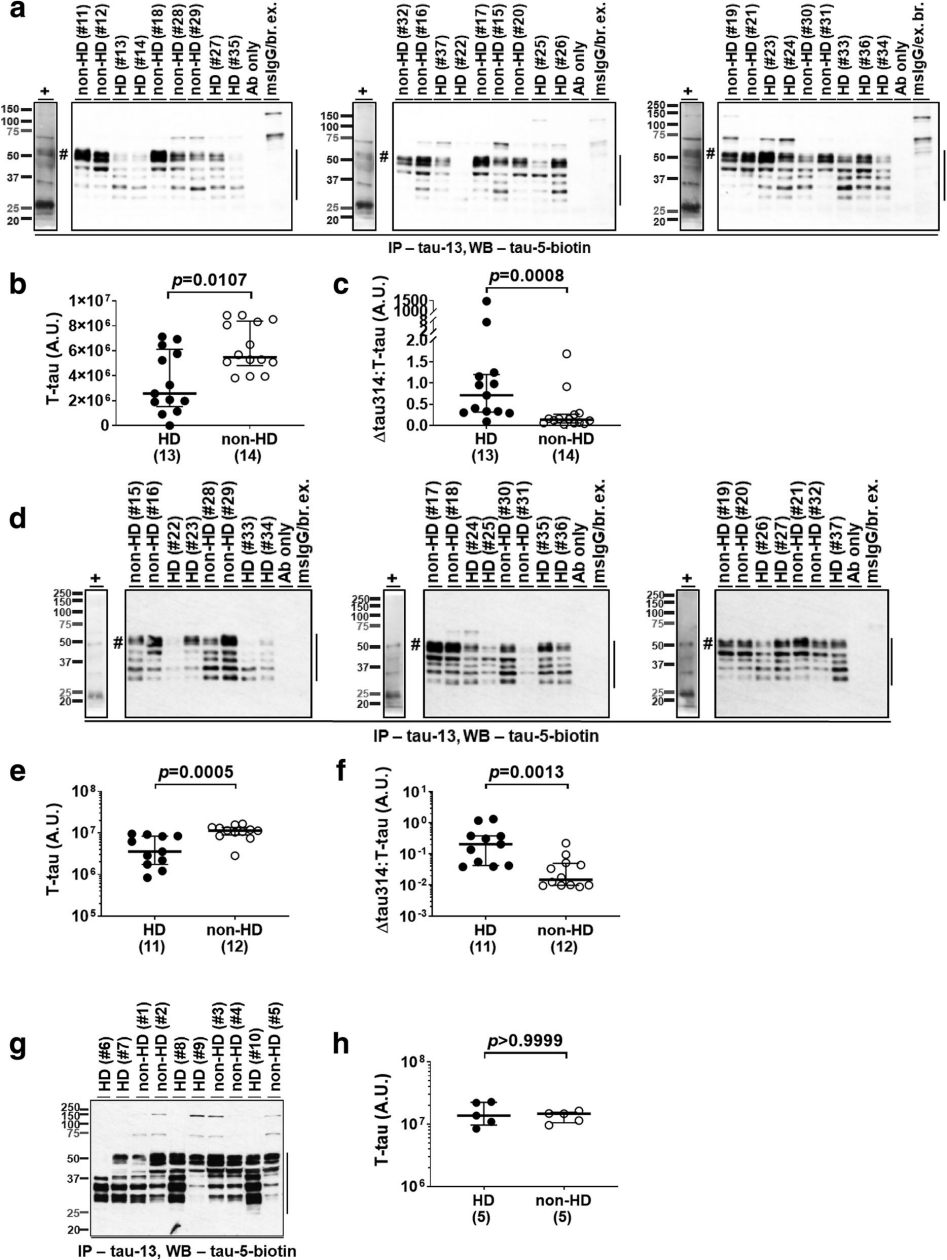
Levels of soluble total tau proteins are lowered in HD patients of the large, but not the small, cohort. **a**, **d**, **g** Representative IP/WB showing that the tau-13-immunoprecipitated soluble total tau (T-tau) proteins (vertical bars) were detected by the biotin-conjugated tau-5 antibody in the prefrontal cortex (Brodmann’s area (BA) 8/9) of subjects from the NIH NeuroBioBank and the New York Brain Bank (**a**), in the caudate nucleus of subjects from the NIH NeuroBioBank (**d**), and in the prefrontal cortex (BA 8) of subjects from the HUB-ICO-IDIBELL Biobank, Spain (**g**). HD, Huntington’s disease patients; non-HD, individuals without Huntington’s disease. +, in vitro synthetic full-length tau protein (hash; 20 μL of sample used in IP, positive control). Sample IDs and disease diagnoses (Additional file 1: Table S1) were shown for IP reactions. Ab only, IP reaction containing only 10 μg of tau-13 (negative control). MsIgG/br. ex., IP reaction containing 10 μg of mouse IgG and 173 μg of soluble brain proteins (negative control). For the MsIgG/br. ex. lanes in figure a, brain extracts were a mixture of the nine samples studied in the same blot with each contributing 1/9th the amount (i.e., 19.2 μg); for the MsIgG/br. ex. lanes in figure d, 1/8th the amount (i.e., 21.6 μg). **b**, **e**, **h** Comparison of levels of T-tau proteins in the prefrontal cortex (BA 8/9) of HD patients and non-HD individuals from the NIH NeuroBioBank and the New York Brain Bank (**b**), in the caudate nucleus, from the NIH NeuroBioBank (**e**), and in the prefrontal cortex (BA 8), from the HUB-ICO-IDIBELL Biobank, Spain (**h**). **c**, **f** Comparison of levels of Δtau314 proteins, normalized to levels of T-tau proteins, in the prefrontal cortex (BA 8/9) of HD patients and non-HD individuals from the NIH NeuroBioBank and the New York Brain Bank (**c**), and in the caudate nucleus, from the NIH NeuroBioBank (**f**). The numbers of analyzed subjects are shown in parentheses. A.U. = arbitrary unit. The y-axes in figures e, f and h are in log scale. Mann-Whitney test was used for between-group comparison; medians (middle long bars), and 1st (lower short bars) and 3rd (upper short bars) quartiles are shown

Interestingly, in the large cohort, levels of T-tau proteins in the prefrontal cortex of HD patients were 53% lower than in non-HD individuals (Fig. 2a and b, Table 3), indicating that the elevated levels of Δtau314 proteins in HD patients were not due to an overall increase in T-tau proteins. The reduction in T-tau proteins in HD patients resulted in 5.3-fold increase in the ratio of Δtau314:T-tau in HD patients versus non-HD individuals (Fig. 2c, Table 3).

Similar to the prefrontal cortex, the levels of T-tau proteins in the caudate nucleus were 69% lower in HD patients than in non-HD individuals (Fig. 2d and e, Table 5). Normalizing Δtau314 to T-tau proteins accentuated their elevated levels, resulting in 13.8-fold higher levels in HD patients compared to the non-HD individuals (Fig. 2f, Table 5). As in the prefrontal cortex, the elevation of Δtau314 proteins in the caudate nucleus of HD patients is not due to an overall increase in T-tau proteins.

In the small cohort, the levels of T-tau proteins were comparable between the HD patients and non-HD individuals (Fig. 2g and h, Additional file 1: Table S3). Our inability to detect a significant decrease in the level of T-tau proteins in the small cohort is likely due to the small sample size (*N* = 5/group). Based on the quantitative analysis of tau 5-reactive proteins in the prefrontal cortex of subjects in the large cohort, we determined that the lowest sample size required to achieve statistical significance under type I error α = 0.05 and power (i.e., (1-β)) = 0.80 is *N* = 82.

To further investigating how levels of Δtau314 proteins associate with levels of soluble T-tau proteins in the large cohort, we measured the levels of T-tau (T-tau (WB)) proteins revealed by direct WB probed with tau-5. These tau-5-detected proteins are not necessarily tau-13-immunoreactive. In the prefrontal cortex, levels of T-tau (WB) proteins in the HD patients were 34% lower than in the non-HD individuals (Additional file 1: Figure S1a and S1b, Table S4). As a result, the ratio of Δtau314:T-tau (WB) in HD patients was 3.8-fold higher than in non-HD individuals (Additional file 1: Figure S2a, Table S4). Similarly, in the caudate nucleus, levels of the T-tau (WB) proteins were 44% lower in HD patients than in non-HD individuals (Additional file 1: Figure S1c and S1d, Table S5). Normalizing Δtau314 to T-tau (WB) proteins resulted in 21.9-fold higher levels in HD patients than in non-HD individuals (Additional file 1: Figure S2b, Table S5). These findings further support that the higher levels of Δtau314 proteins in HD patients are not due to an increase in T-tau proteins.

To confirm that our findings in levels of Δtau314 and T-tau proteins are not due to difference in the amount of soluble brain proteins applied to IP/WB, we analyzed levels of the housekeeping protein GAPDH. We showed that the levels of GAPDH were comparable between HD patients and non-HD individuals in both prefrontal cortex and caudate nucleus (Additional file 1: Figure S3, Tables 3 and 5).

Lastly, we examined the relationships between demographic characteristics of subjects and levels of Δtau314 proteins in both the large and small cohorts. We found no apparent correlation of Δtau314 protein levels (normalized to levels of T-tau) with either ages at death or PMIs of brain tissue harvest (Additional file 1: Figure S4a, S4b, S4d, S4e, S4 g and S4 h). Also, normalized Δtau314 protein levels were comparable between female and male individuals (Additional file 1: Figure S4c, S4f and S4i).

### Levels of Casp2 are higher in the prefrontal cortex and caudate nucleus of HD patients

To understand the extent to which Casp2 is involved in production of Δtau314 proteins in HD, we measured levels of Casp2 detected by a monoclonal anti-Casp2 antibody directed against a C-terminal epitope (Table 1) in the prefrontal cortex and the caudate nucleus of subjects in the large cohort. Prior to the experiments, we validated the binding specificity of the anti-Casp2 antibody to the cleaved small subunit of recombinant Casp2 (data not shown).

**Fig. 3:**
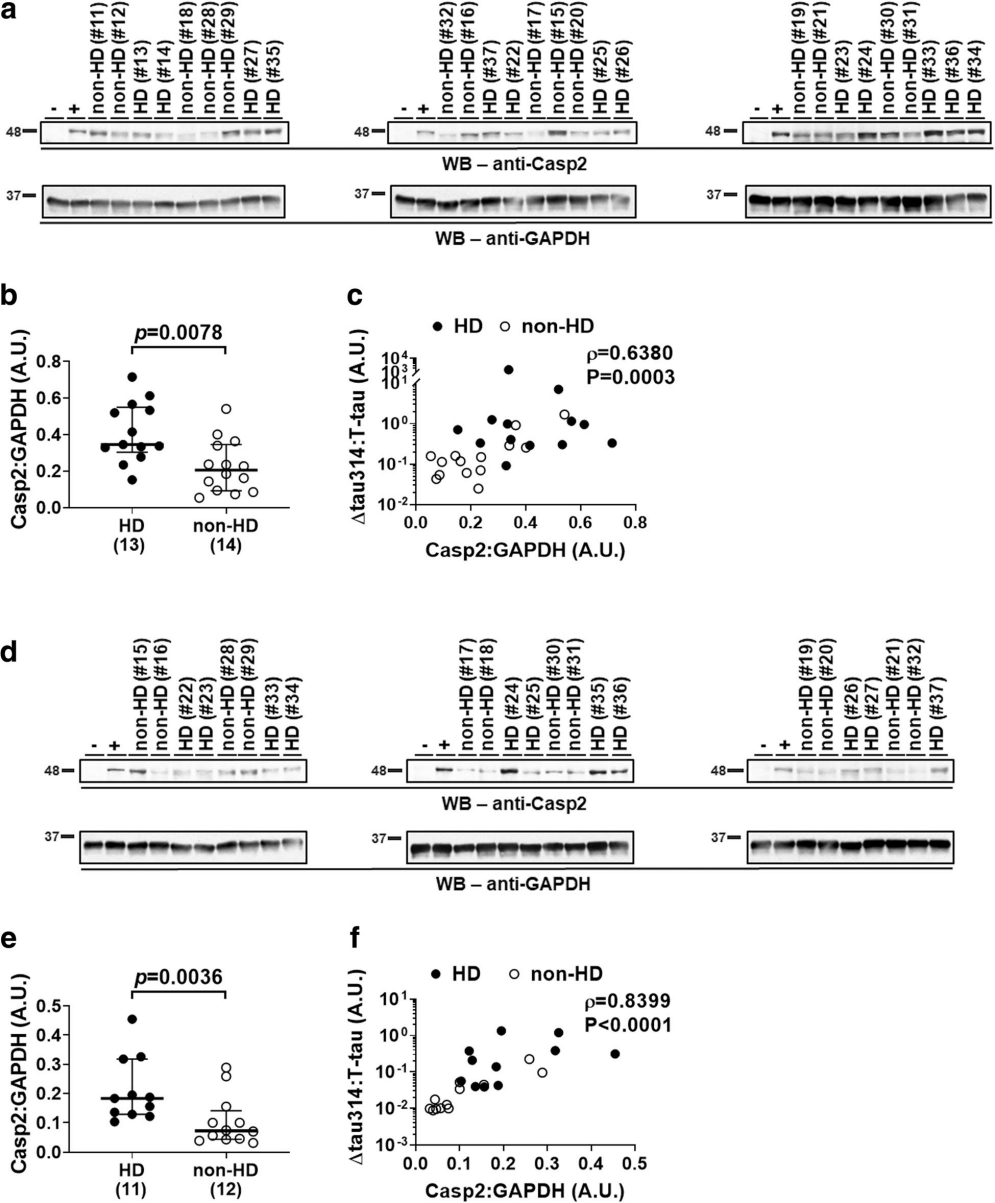
Levels of Casp2 are higher in HD patients of the large cohort. **a**, **d** Representative WB showing that Casp2 (~ 48-kDa full-length form, upper panels) and GAPDH (loading control, lower panels; revealed in the same blots as Casp2) were detected in the prefrontal cortex (Brodmann’s area (BA) 8/9) of subjects from the NIH NeuroBioBank and the New York Brain Bank (**a**), and in the caudate nucleus of subjects from the NIH NeuroBioBank (**d**). HD, Huntington’s disease patients; non-HD, individuals without Huntington’s disease. -, Casp2 knock-out FVB/129S6 mice (negative control); +, wild-type non-transgenic FVB/129S6 mice (positive control). Sample IDs and disease diagnoses (Additional file 1: Table S1) were shown. **b**, **e** Comparison of levels of Casp2 in the prefrontal cortex (BA 8/9) of HD patients and non-HD individuals from the NIH NeuroBioBank and the New York Brain Bank (**b**), and in the caudate nucleus, from the NIH NeuroBioBank (**e**). The numbers of analyzed subjects are shown in parentheses. Mann-Whitney test was used for between-group comparison; medians (middle long bars), and 1st (lower short bars) and 3rd (upper short bars) quartiles are shown. **c**, **f** Correlation of levels of Casp2 (normalized to the house-keeping protein GAPDH) and levels of Δtau314 proteins (normalized to levels of T-tau proteins) in the prefrontal cortex (BA 8/9) of HD patients and non-HD individuals from the NIH NeuroBioBank and the New York Brain Bank (**c**), and in the caudate nucleus, from the NIH NeuroBioBank (**f**). Spearman’s rank-order correlation was used. The y-axes in figures c and f are in log scale. A.U. = arbitrary unit

In the prefrontal cortex, levels of Casp2 proteins, normalized to levels of the house-keeping protein GAPDH, were 1.7-fold higher in HD patients than in non-HD individuals (Fig. 3a and b, Table 3). In the caudate nucleus, levels of Casp2 proteins, normalized to levels of GAPDH, were 2.5-fold higher in HD patients than in non-HD individuals (Fig. 3d and e, Table 5). The finding that levels of both Δtau314 and Casp2 proteins are higher in HD patients than in non-HD individuals, in two distinct HD-affected brain structures, supports the active involvement of Casp2 in Δtau314 production in human subjects, particularly in HD patients.

In addition, levels of Casp2 (normalized to levels of GAPDH) correlated well with levels of Δtau314 (normalized to levels of T-tau) in both the prefrontal cortex (Fig. 3c) and the caudate nucleus (Fig. 3f), which further supports an association between Casp2 and the production of Δtau314 proteins.

## Discussion

In this study, we focused on understanding the potential role of Δtau314, a soluble, cognition-related tau protein species associated with Casp2, in HD. Using biochemical analyses, we detected Δtau314 proteins in the striatum (caudate nucleus) and prefrontal cortex (BA 8/9), areas that are especially vulnerable to HD pathology. We showed that levels of Δtau314 proteins are 1.6- to 5.6-fold higher in both regions in HD patients compared to non-HD individuals, that levels of Casp2 are 1.7- to 2.5-fold higher in HD patients than in non-HD individuals, and that levels of Casp2 and Δtau314 proteins are correlated in both studied brain structures. Our results are supported by a previous finding that the immunostaining intensity of Casp2, which catalyzes Δtau314 production, is more intense in degenerating caudate neurons of HD patients than in those of age-matched controls [16]. Also of note, ~ 35- and ~ 40- kDa tau protein species that match the migration pattern of some of the Δtau314 proteins in SDS-PAGE show a remarkably higher level in the cortex of HD patients than control individuals [11], though their exact identities are unclear in that study.

We used IP/WB, rather than WB only, for identifying Δtau314 in soluble human brain homogenates because of the enhanced sensitivity and specificity. Our choice of using monoclonal antibody tau-13 as the capture reagent for immunoprecipitation in this study are based on two considerations: 1) the sensitivity of tau-13, and 2) the relevance of measured proteins to cognitive dysfunction and dementia. Specifically, we tried several widely used, commercially available antibodies, including mouse monoclonal tau-5, rabbit polyclonal K9JA, in addition to tau-13, to immunoprecipitate tau proteins from soluble human brain homogenates. We found that tau-13 produced the most sensitive results. For example, the ~ 45-kDa Δtau314 protein that is derived from the longest tau isoform (2N4R) can only be detected using tau-13 as the capture reagent. In addition, since Casp2-catalyzed production of the *tau-13-immunoreactive* Δtau314 mediates synaptotoxicity in cellular models and cognition-impairing effects in mice, and levels of the *tau-13-immunoprecipitated* Δtau314 proteins are higher in subjects with mild cognitive impairment and AD than cognitively normal individuals [44], we focused on *tau-13-immunoprecipitated* Δtau314 proteins for this study. It is possible that other Δtau314 species exist in the brain, and that N-terminally truncated Δtau314 proteins may also contribute to the pathogenesis of neurodegenerative diseases.

Δtau314 proteins are among the reported post-translationally modified tau species whose levels are elevated in AD subjects, compared to healthy individuals, and correlate with the severity of cognitive dysfunction in a variety of mouse lines modeling frontotemporal dementia [24, 43, 44]. Recently, we found higher levels of Δtau314 proteins in patients with Lewy body dementia than in non-demented Parkinson’s disease patients (Smith et al. *Acta Neuropathol. Commun.* in press). These findings, taken with the current results, suggest that dementia may depend on Casp2-cleavage of tau in several neurodegenerative conditions.

We found elevated Δtau314 protein levels not only in the prefrontal cortex but also in the caudate nucleus of HD patients, a brain structure known to be involved in motor control, as well as some forms of cognition. Thus, whether Casp2-mediated Δtau314 production plays a role in a variety of behavioral abnormalities—both related and unrelated to cognition—in HD is an important question to be addressed in future studies.

Our findings of lower total tau levels in HD are superficially inconsistent with a previous finding of increased total tau levels in the frontal cortex of HD patients [11]. This discrepancy is likely due to different experimental methods in the two studies. Specifically, detergent-containing brain extracts were used in the previous study, while detergent-free, aqueous brain extracts were used in the present study. The detergent extracts likely contained solubilized tau aggregates, which would be absent in the aqueous extracts. In addition, monoclonal 5E2 antibody-reactive, ~ 49-kDa tau proteins were quantified in the previous study, whereas tau-13-immunoprecipitated, tau-5-reactive, ~ 30–55-kDa tau proteins were quantified in this study.

The number of CAG repeats has profound implications in both clinical phenotype and pathologic characteristics of HD [2, 29], and its relationship to the level of soluble Δtau314 proteins warrants further investigation. Specifically, it would be interesting to extend the detection and quantitative analysis of Δtau314 proteins to juvenile-onset HD patients, a population that often has significantly larger numbers of CAG repeats than the adult-onset patients and whose cognitive abilities are significantly impaired [27, 30]. In this study, however, we chose to study HD subjects of 50 years or older at the age of death to avoid potential juvenile-onset HD-associated confounding factors since adult- and juvenile- onset HD patients manifest distinct clinical, pathologic and biochemical features [4, 5, 37], suggesting different pathophysiological mechanisms through which mHTT exerts toxic effects.

Our finding of the association of HD with elevated levels of Δtau314 supports the potential use of Casp2 inhibitors to ameliorate neurological dysfunction in HD. Broad-spectrum inhibitors of caspases were shown to reduce toxicity in cellular models of HD [1, 20, 42], and ameliorate behavioral abnormalities in HD mice [28], indicating the disease-modifying effects of pharmacological blockage of caspase activities. In addition, using in silico structure-based design coupled with solid-state synthesis, a group of pentapeptide aldehyde compounds were recently developed, and some showed high affinity (IC_50_ in nanomolar magnitude) and selectivity (60-fold difference in IC_50_ between Casp2 and caspase-3) to Casp2 [22], drawing attention to the possibility of targeting Casp2 activity as a therapeutic strategy in HD.

There are several limitations to this study. First, we did not determine the precise numbers of CAG repeats in the subjects, and thus the relationship between the degree of CAG expansion and levels of Δtau314 (and T-tau) proteins is unknown. Second, to better understand the association of Δtau314 proteins with other neurological functions, it will be necessary to perform biochemical analyses of Δtau314 proteins in other brain structures (e.g., hippocampus and cerebellum). Third, an important question concerns the extent to which levels of Δtau314 proteins correlate with the severity of cognitive dysfunction. For the subjects of the large cohort study, eight of the thirteen HD patients had cognition assessment records, and all demonstrated cognitive deficits prior to death. Meanwhile, four of the fourteen non-HD individuals had cognition assessment records, and all demonstrated normal cognitive function in their last assessment (Additional file 1: Table S1). Despite these observations, we did not perform a comprehensive evaluation of the correlation between levels of Δtau314 proteins and the severity of cognitive deficits, because cognitive function assessments were lacking or not quantitatively provided for the other subjects, and different assessment approaches were used for those subjects whose cognitive functions were quantitatively measured. This is a question that may be addressed in humans and in animal models of HD in future studies. Fourth, the Casp2 protein band that we quantified using WB does not represent the fully active form. We identified only the ~ 48-kDa protein band, likely representing the procaspase-2 form, which, upon dimerization, shows a partial catalytic activity [3]. The fact that no cleaved small subunit of Casp2 was detected in the studied brains suggests that the fully active Casp2 may constitute a minute portion of the total pool of Casp2, and levels of the fully active Casp2 are below the limit of detection of WB. To the authors’ knowledge, assays that specifically measure Casp2 activity in the brain are not yet available; development of such an assay is a priority of our future studies.

## Conclusions

In conclusion, Δtau314 proteins are present in the prefrontal cortex and caudate nucleus of human subjects, levels of Casp2 and Δtau314 proteins are correlated, and levels of both Casp2 and Δtau314 proteins are elevated in HD patients, compared to non-HD individuals. Findings of this study extend our understanding of the contribution of Casp2-mediated tau cleavage to HD pathogenesis, and support the development and exploration of Casp2 inhibitors in the treatment of HD.

## Additional file

Additional file 1: **Figure S1.** Levels of tau-5-reactive soluble proteins revealed by direct Western blotting are lowered in HD patients than non-HD individuals. **Figure S2.** Levels of tau-13-immunoreactive Δtau314 proteins, following normalization to levels of tau-5-reactive proteins (T-tau (WB)) revealed by direct Western blotting, are higher in HD patients than non-HD individuals. **Figure S3.** Levels of glyceraldehyde 3-phosphate dehydrogenase (GAPDH) are comparable between HD patients and non-HD individuals of the large cohort. **Figure S4.** The relationships of Δtau314 protein levels with demographic characteristics of subjects.** Table S1.** Demographic and neuropathological characteristics of human subjects. **Table S2.** Comparison of demographic characteristics of HD patients and non-HD individuals from the HUB-ICO-IDIBELL Biobank, Spain used in the study of proteins in the prefrontal cortex (BA8). **Table S3.** A statistical comparison of protein levels of HD patients and non-HD individuals from the HUB-ICO-IDIBELL Biobank, Spain. **Table S4.** A statistical comparison of levels of proteins revealed by direct Western blotting (WB) probed with tau-5 antibody in the prefrontal cortex (BA8/9) of HD patients and non-HD individuals from the NIH NeuroBioBank and the New York Brain Bank. **Table S5.** A statistical comparison of protein levels of proteins revealed by direct Western blotting (WB) probed with tau-5 antibody in the caudate nucleus of HD patients and non-HD individuals from the NIH NeuroBioBank. File S1 Supplementary references. (ZIP 8370 kb)

## Data Availability

All data generated or analyzed during this study are included in this published article and its supplementary information files.

## Abbreviations

0N4R: (microtubule-associated protein tau isoform containing) none N-terminal inserts encoded by exons 2 and 3 and four microtubule-binding repeats
2N4R: (microtubule-associated protein tau isoform containing) two N-terminal inserts encoded by exons 2 and 3 and four microtubule-binding repeats
AD: Alzheimer’s disease
BA: Brodmann’s area
Casp2: Caspase-2
caspase: Cysteine-dependent aspartate-directed protease
cDNA: Complementary DNA
D314: aspartate being the 314th amino acid residue of a protein/polypeptide
EDTA: Ethylenediaminetetraacetic acid
GAPDH: Glyceraldehyde 3-phosphate dehydrogenase
HD: Huntington’s disease
HRP: Horseradish peroxidase
HTT: Huntingtin
IC_50_: Half maximal inhibitory concentration
IgG: Immunoglobulin G
IP: Immunoprecipitation
IRBs: Institutional Review Boards
*IT15*: *Interesting transcript 15*
kDa: kilo-Dalton
*MAPT*: Human microtubule-associated protein tau gene
MAPT: Microtubule-associated protein tau
*Mapt*: Mouse microtubule-associated protein tau gene
MCI: Mild cognitive impairment
mHTT: Mutant huntingtin
NIH: National Institutes of Health
NYBB: New York Brain Bank
PAGE: Polyacrylamide gel electrophoresis
PCR: Polymerase chain reaction
PMI: Post-mortem interval
rTg4510: transgenic mouse line 129S6.Cg-Tg(Camk2a-tTA)1Mmay/JlwsJ; Fgf14Tg(tetO-MAPT*P301L)4510Kha/J, which overexpresses human microtubule-associated protein tau with the P301L mutation associated with frontotemporal dementia with parkinsonism linked to chromosome 17
SDS: Sodium dodecyl sulfate
Tris–HCl: Tris(hydroxymethyl)aminomethane-hydrochloric acid
Triton X-100: polyethylene glycol p-(1,1,3,3-tetramethylbutyl)-phenyl ether
T-tau: soluble total tau that is immunoprecipitated by the monoclonal antibody tau-13 directed against tau a.a. 15–25 (tau 2N4R numbering system) and immunoreactive to the monoclonal antibody tau-5 directed against tau a.a. 210–241 (tau 2N4R numbering system)
Tween 20: polyoxyethylene (20) sorbitan monolaurate
v/v: volume/volume
w/v: Weight/volume
WB: Western blotting
Δtau314: a C-terminally truncated tau protein ending at the 314th amino acid residue (aspartate) of the longest human tau splicing isoform
