# Supplementary material for: A soluble truncated tau species related to cognitive dysfunction and caspase-2 is elevated in the brain of Huntington’s disease patients
Source: Acta Neuropathol Commun. 2019 Jul 30;7:111. doi: 10.1186/s40478-019-0764-9 (PMC6664763; doi:10.1186/s40478-019-0764-9)
Supplement: Supplementary file 1 — Figure S1. Levels of tau-5-reactive soluble proteins revealed by direct Western blotting are lowered in HD patients than non-HD individuals. Figure S2. Levels of tau-13-immunoreactive Δtau314 proteins, following normalization to levels of tau-5-reactive proteins (T-tau (WB)) revealed by direct Western blotting, are higher in HD patients than non-HD individuals. Figure S3. Levels of glyceraldehyde 3-phosphate dehydrogenase (GAPDH) are comparable between HD patients and non-HD individuals of the large cohort. Figure S4. The relationships of Δtau314 protein levels with demographic characteristics of subjects. Table S1. Demographic and neuropathological characteristics of human subjects. Table S2. Comparison of demographic characteristics of HD patients and non-HD individuals from the HUB-ICO-IDIBELL Biobank, Spain used in the study of proteins in the prefrontal cortex (BA8). Table S3. A statistical comparison of protein levels of HD patients and non-HD individuals from the HUB-ICO-IDIBELL Biobank, Spain. Table S4. A statistical comparison of levels of proteins revealed by direct Western blotting (WB) probed with tau-5 antibody in the prefrontal cortex (BA8/9) of HD patients and non-HD individuals from the NIH NeuroBioBank and the New York Brain Bank. Table S5. A statistical comparison of protein levels of proteins revealed by direct Western blotting (WB) probed with tau-5 antibody in the caudate nucleus of HD patients and non-HD individuals from the NIH NeuroBioBank. File S1 Supplementary references. (ZIP 8370 kb) [file 40478_2019_764_MOESM1_ESM.zip › Supporting information.docx]

Supporting information

**A soluble truncated tau species related to cognitive dysfunction and caspase-2 is elevated in the brain of Huntington’s disease patients**

Peng Liu, Benjamin R. Smith, Eric S. Huang, Abhishek Mahesh, Jean Paul G. Vonsattel, Ashley J. Petersen, Rocio Gomez-Pastor, Karen H. Ashe

This document contains four supplementary figures, five supplementary tables and one supplementary reference.

**Figure S1. Levels of tau-5-reactive soluble proteins revealed by direct Western blotting are lowered in HD patients than non-HD individuals.** (**a, c**) Representative Western blots (WBs) showing that soluble tau proteins were detected by the biotin-conjugated tau-5 antibody in the prefrontal cortex (Brodmann’s area (BA) 8/9) of subjects from the NIH NeuroBioBank and the New York Brain Bank (a), and in the caudate nucleus of subjects from the NIH NeuroBioBank (c). HD, Huntington’s disease patients; non-HD, individuals without Huntington’s disease. +, non-transgenic mouse (FVB/129S6) brain extracts (positive control). Sample IDs and disease diagnoses were shown. For each lane, 100 µg of water-soluble brain extracts were loaded. (**b, d**) Comparison of levels of tau-5-reactive proteins (T-tau (WB), shown within brackets of figures a and c) in the prefrontal cortex (BA 8/9) of HD patients and non-HD individuals from the NIH NeuroBioBank and the New York Brain Bank (b), and in the caudate nucleus, from the NIH NeuroBioBank (d). The numbers of analyzed subjects are shown in parentheses. A.U. = arbitrary unit. Mann-Whitney test was used for between-group comparison; medians (middle long bars), and 1^st^ (lower short bars) and 3^rd^ (upper short bars) quartiles are shown.

**Figure S2. Levels of tau-13-immunoreactive Δtau314 proteins, following normalization to levels of tau-5-reactive proteins (T-tau (WB)) revealed by direct Western blotting, are higher in HD patients than non-HD individuals.** (**a, b**) Comparison of normalized levels of Δtau314 proteins in the prefrontal cortex (BA 8/9) of HD patients and non-HD individuals from the NIH NeuroBioBank and the New York Brain Bank (a), and in the caudate nucleus, from the NIH NeuroBioBank (b). HD, Huntington’s disease patients; non-HD, individuals without Huntington’s disease. The numbers of analyzed subjects are shown in parentheses. A.U. = arbitrary unit. The y-axes are in log scale. Mann-Whitney test was used for between-group comparison; medians (middle long bars), and 1^st^ (lower short bars) and 3^rd^ (upper short bars) quartiles are shown.

**Figure S3. Levels of glyceraldehyde 3-phosphate dehydrogenase (GAPDH) are comparable between HD patients and non-HD individuals of the large cohort.** (**a**, **c**) Representative WB showing that GAPDH were detected in the prefrontal cortex (Brodmann’s area 8/9) (a), and the caudate nucleus (c). HD, Huntington’s disease patients; non-HD, individuals without Huntington’s disease. +, GAPDH of aqueous rTg4510 mouse forebrain extracts (20 µg of total brain protein loaded, positive control). Sample IDs and disease diagnoses (Table S1) were shown above blots. X, empty lanes. (**b**, **d**) Comparison of levels of GAPDH in the prefrontal cortex (b) and the caudate nucleus (d) of HD patients and non-HD individuals. The numbers of analyzed subjects are shown in parentheses. A.U. = arbitrary unit. The y-axes in figures are in log scale. Mann-Whitney test was used for between-group comparison; medians (middle long bars), and 1^st^ (lower short bars) and 3^rd^ (upper short bars) quartiles are shown.

**Figure S4. The relationships of Δtau314 protein levels with demographic characteristics of subjects.** (**a**) The correlation of Δtau314 protein levels (normalized to levels of soluble total tau (T-tau) proteins) in the prefrontal cortex (Brodmann’s area (BA) 8) to ages at death of subjects from the HUB-ICO-IDIBELL Biobank, Spain. (**b**) The correlation of Δtau314 protein levels (normalized to levels of T-tau proteins) in the prefrontal cortex (BA 8) to post-mortem intervals (PMIs) of subjects from the HUB-ICO-IDIBELL Biobank, Spain. (**c**) The levels of Δtau314 proteins (normalized to levels of T-tau proteins) in the prefrontal cortex (BA 8) of the female individuals from the HUB-ICO-IDIBELL Biobank, Spain are not significantly different from those of the male individuals. (**d**) The correlation of Δtau314 protein levels (normalized to levels of T-tau proteins) in the prefrontal cortex (BA 8/9) to ages at death of subjects from the National Institutes of Health (NIH) NeuroBioBank and the New York Brain Bank (NYBB) at Columbia University, New York City, New York. (**e**) The correlation of Δtau314 protein levels (normalized to levels of T-tau proteins) in the prefrontal cortex (BA 8/9) to PMIs of subjects from the NIH NeuroBioBank and the NYBB. (**f**) The levels of Δtau314 proteins (normalized to levels of T-tau proteins) in the prefrontal cortex (BA 8/9) of the female individuals from the NIH NeuroBioBank and the NYBB are not significantly different from those of the male individuals. (**g**) The correlation of Δtau314 protein levels (normalized to levels of T-tau proteins) in the caudate nucleus to ages at death of subjects from the NIH NeuroBioBank. (**h**) The correlation of Δtau314 protein levels (normalized to levels of T-tau proteins) in the caudate nucleus to PMIs of subjects from the NIH NeuroBioBank. (**i**) The levels of Δtau314 proteins (normalized to levels of T-tau proteins) in the caudate nucleus of the female individuals from the NIH NeuroBioBank are not significantly different from those of the male individuals. Notably, all figures are in log scale. The numbers of analyzed subjects in figures c, f and i are shown in parentheses. For figures a, b, d, e, g and h, Spearman’s rank-order correlation was used. For figures c, f and i, two-tailed, unpaired Mann-Whitney test was used; medians (middle long bars), and 1^st^ (lower short bars) and 3^rd^ (upper short bars) quartiles are shown. A.U. = arbitrary unit.

**Table S1 Demographic and neuropathological characteristics of human subjects**

| **ID** | **Diagnosis^a^** | **Age (yr)^b^** | **Sex^c^** | **PMI (hr)^d^** | **Neuropathology diagnosis^e^** | **Cognitive function assessment** | **Source** |
| --- | --- | --- | --- | --- | --- | --- | --- |
| 1 | non-HD | 73 | F | 7 | N/A | N/A | HUB-ICO-IDIBELL Biobank, Spain |
| 2 | non-HD | 73 | F | 5.5 | N/A | N/A |  |
| 3 | non-HD | 51 | M | 4 | N/A | N/A |  |
| 4 | non-HD | 24 | F | 6 | N/A | N/A |  |
| 5 | non-HD | 46 | M | 15 | N/A | N/A |  |
| 6 | HD | 72 | F | 7 | VG4 | N/A |  |
| 7 | HD | 72 | F | 5 | VG4 | N/A |  |
| 8 | HD | 68 | M | 4 | VG4 | N/A |  |
| 9 | HD | 28 | F | 4.2 | VG4 | N/A |  |
| 10 | HD | 60 | M | 13.1 | VG4 | N/A |  |
| 11 | non-HD | 57 | F | 11.63 | N/A | normal cognitive function | New York Brain Bank |
| 12 | non-HD | 57 | M | 11.63 | N/A | normal cognitive function |  |
| 13 | HD | 66 | F | 6 | VG4 | severely demented at the age of death; Mini-mental status was 52/57 in 1993 at age 47 |  |
| 14 | HD | 64 | F | 20.32 | VG4 | severely demented at the age of death |  |
| 15 | non-HD | 62 | F | 18.9 | N/A | N/A | NIH NeuroBioBank |
| 16 | non-HD | 60 | F | 20.5 | N/A | N/A |  |
| 17 | non-HD | 68 | F | 19.1 | N/A | N/A |  |
| 18 | non-HD | 68 | M | 10.8 | N/A | N/A |  |
| 19 | non-HD | 71 | F | 16.4 | N/A | 30/30 (last MMSE^f^ score) = normal, 14 months prior to death; CDR^g^ = 0 |  |
| 20 | non-HD | 77 | M | 14.7 | N/A | N/A |  |
| 21 | non-HD | 79 | F | 17.8 | N/A | 38/50 (last TICS-M^h^ score) = normal; 7 months prior to death |  |
| 22 | HD | 62 | F | 14.7 | UNKNOWN | 24/30 (last MoCA^i^ score) = MCI, 3 years prior to death |  |
| 23 | HD | 73 | F | 15.7 | UNKNOWN | N/A |  |
| 24 | HD | 68 | F | 12.2 | VG1 | CDR^g^ = 3 |  |
| 25 | HD | 58 | F | 18.7 | VG3 | CDR^g^ = 3 |  |
| 26 | HD | 76 | M | 21.7 | VG3 | 23/30 (last MMSE^f^ score) = MCI, 3 years prior to death |  |
| 27 | HD | 63 | M | 8.6 | VG4 | N/A |  |
| 28 | non-HD | 89 | M | 9 | N/A | N/A |  |
| 29 | non-HD | 81 | F | 11.3 | N/A | N/A |  |
| 30 | non-HD | 82 | F | 12.9 | N/A | N/A |  |
| 31 | non-HD | 72 | M | 12.2 | N/A | N/A |  |
| 32 | non-HD | 76 | M | 11 | N/A | N/A |  |
| 33 | HD | 50 | M | 12 | VG2 | loss of short term memory prior to death |  |
| 34 | HD | 71 | F | 12 | UNKNOWN | At age 63, quite prominent problems with cognition, including some difficulties finding her way around her own neighborhood and if places are not familiar to her. |  |
| 35 | HD | 77 | F | 12 | VG3-4 | N/A |  |
| 36 | HD | 56 | F | 9.2 | VG2-3 | N/A |  |
| 37 | HD | 65 | M | 9.1 | VG2 | N/A |  |

^a^HD = Huntington’s disease; ^b^Age = age at death, yr = year; ^c^F = female, M = male; ^d^PMI = post-mortem interval, hr = hour; ^e^A grading system [1] was applied used to assess the striatal neuropathology of post-mortem HD patients; ^f^MMSE = Mini-Mental State Examination; ^g^CDR = Clinical Dementia Rating; ^h^TICS-M = Modified Telephone Interview for Cognitive Status; ^i^MoCA = Montreal Cognitive Assessment; N /A = not applicable.

**Table S2 Comparison of demographic characteristics of HD patients and non-HD individuals from the HUB-ICO-IDIBELL Biobank, Spain used in the study of proteins in the prefrontal cortex (BA8)**

|  | **non-HD** | **HD** | ***p* value** |
| --- | --- | --- | --- |
| **Sample size, *N*** | 5 | 5 |  |
| **Age at death [yr]:**  **median (1^st^ quartile, 3^rd^ quartile)**  **range** | 51 (35, 73)  24-73 | 68 (44, 72)  28-72 | 0.98^a^ |
| **Sex, female/male, No. (% female)** | 3/2 (60%) | 3/2 (60%) | >0.99^b^ |
| **Post-mortem interval [hr]:**  **median (1^st^ quartile, 3^rd^ quartile)**  **range** | 6.0 (4.8, 11.0)  4.0-15.0 | 5.0 (4.1, 10.1)  4.0-13.1 | 0.63^a^ |

^a^Two-tailed, unpaired Mann-Whitney test.

^b^Two-sided Fisher’s exact test.

**Table S3 A statistical comparison of protein levels of HD patients and non-HD individuals from the HUB-ICO-IDIBELL Biobank, Spain**

|  | **Mann-Whitney^a^** | ***t*-test^b^** | **Multiple linear regression^c^** |
| --- | --- | --- | --- |
| **Δtau314** | *p*=0.016 | *p*=0.039 | *p*=0.072 |
| **T-tau** | *p*>0.99 | *p*=0.68 | *p*=0.86 |
| **Δtau314:T-tau** | *p*=0.15 | *p*=0.18 | *p*=0.20 |

^a^The two-tailed, unpaired Mann-Whitney test was performed.

^b^The two-tailed, unpaired *t*-test with Welch’s correction was performed on the log transformed outcomes.

^c^Multiple linear regression was used to analyze the log transformed outcomes of protein levels with adjustment for age at death, sex, and PMI of brain tissue harvest.

**Table S4 A statistical comparison of levels of proteins revealed by direct Western blotting (WB) probed with tau-5 antibody in the prefrontal cortex (BA8/9) of HD patients and non-HD individuals from the NIH NeuroBioBank and the New York Brain Bank**

|  | **Mann-Whitney^a^** | ***t*-test^b^** | **Multiple linear regression^c^** |
| --- | --- | --- | --- |
| **T-tau (WB)** | *p*=0.00053 | *p*=0.0050 | *p*=0.0023 |
| **Δtau314:T-tau (WB)** | *p*=0.0015 | *p*=0.0024 | *p*=0.0051 |

^a^The two-tailed, unpaired Mann-Whitney test was performed.

^b^The two-tailed, unpaired *t*-test with Welch’s correction was performed on the log transformed outcomes.

^c^Multiple linear regression was used to analyze the log transformed outcomes of protein levels with adjustment for age at death, sex, and PMI of brain tissue harvest.

**Table S5 A statistical comparison of protein levels of proteins revealed by direct Western blotting (WB) probed with tau-5 antibody in the caudate nucleus of HD patients and non-HD individuals from the NIH NeuroBioBank**

|  | **Mann-Whitney^a^** | ***t*-test^b^** | **Multiple linear regression^c^** |
| --- | --- | --- | --- |
| **T-tau (WB)** | *p*=0.0056 | *p*=0.018 | *p*=0.16 |
| **Δtau314:T-tau (WB)** | *p*=0.0045 | *p*=0.0027 | *p*=0.021 |

^a^The two-tailed, unpaired Mann-Whitney test was performed.

^b^The two-tailed, unpaired *t*-test with Welch’s correction was performed on the log transformed outcomes.

^c^Multiple linear regression was used to analyze the log transformed outcomes of protein levels with adjustment for age at death, sex, and PMI of brain tissue harvest.

**Supplementary references**

1. Vonsattel JP, Myers RH, Stevens TJ, Ferrante RJ, Bird ED, Richardson EP, Jr.: **Neuropathological classification of Huntington's disease.** *J Neuropathol Exp Neurol* 1985, **44:**559-577.
