# Supplementary figures and images for: A soluble truncated tau species related to cognitive dysfunction and caspase-2 is elevated in the brain of Huntington’s disease patients
Source: Acta Neuropathol Commun. 2019 Jul 30;7:111. doi: 10.1186/s40478-019-0764-9 (PMC6664763; doi:10.1186/s40478-019-0764-9)

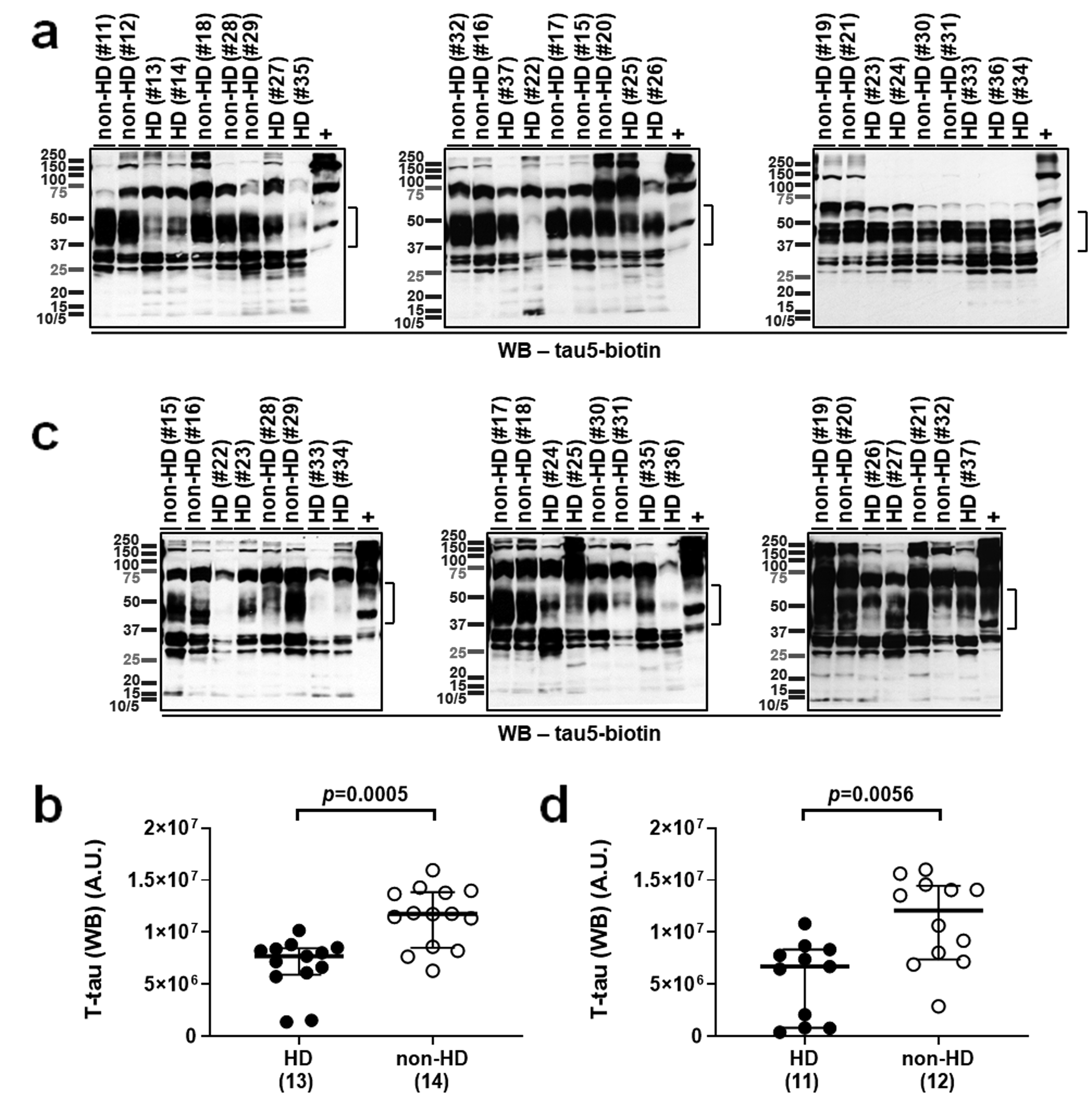

Supplement: Supplementary file 1 — Figure S1. Levels of tau-5-reactive soluble proteins revealed by direct Western blotting are lowered in HD patients than non-HD individuals. Figure S2. Levels of tau-13-immunoreactive Δtau314 proteins, following normalization to levels of tau-5-reactive proteins (T-tau (WB)) revealed by direct Western blotting, are higher in HD patients than non-HD individuals. Figure S3. Levels of glyceraldehyde 3-phosphate dehydrogenase (GAPDH) are comparable between HD patients and non-HD individuals of the large cohort. Figure S4. The relationships of Δtau314 protein levels with demographic characteristics of subjects. Table S1. Demographic and neuropathological characteristics of human subjects. Table S2. Comparison of demographic characteristics of HD patients and non-HD individuals from the HUB-ICO-IDIBELL Biobank, Spain used in the study of proteins in the prefrontal cortex (BA8). Table S3. A statistical comparison of protein levels of HD patients and non-HD individuals from the HUB-ICO-IDIBELL Biobank, Spain. Table S4. A statistical comparison of levels of proteins revealed by direct Western blotting (WB) probed with tau-5 antibody in the prefrontal cortex (BA8/9) of HD patients and non-HD individuals from the NIH NeuroBioBank and the New York Brain Bank. Table S5. A statistical comparison of protein levels of proteins revealed by direct Western blotting (WB) probed with tau-5 antibody in the caudate nucleus of HD patients and non-HD individuals from the NIH NeuroBioBank. File S1 Supplementary references. (ZIP 8370 kb) [file 40478_2019_764_MOESM1_ESM.zip › Figure S1.tif]

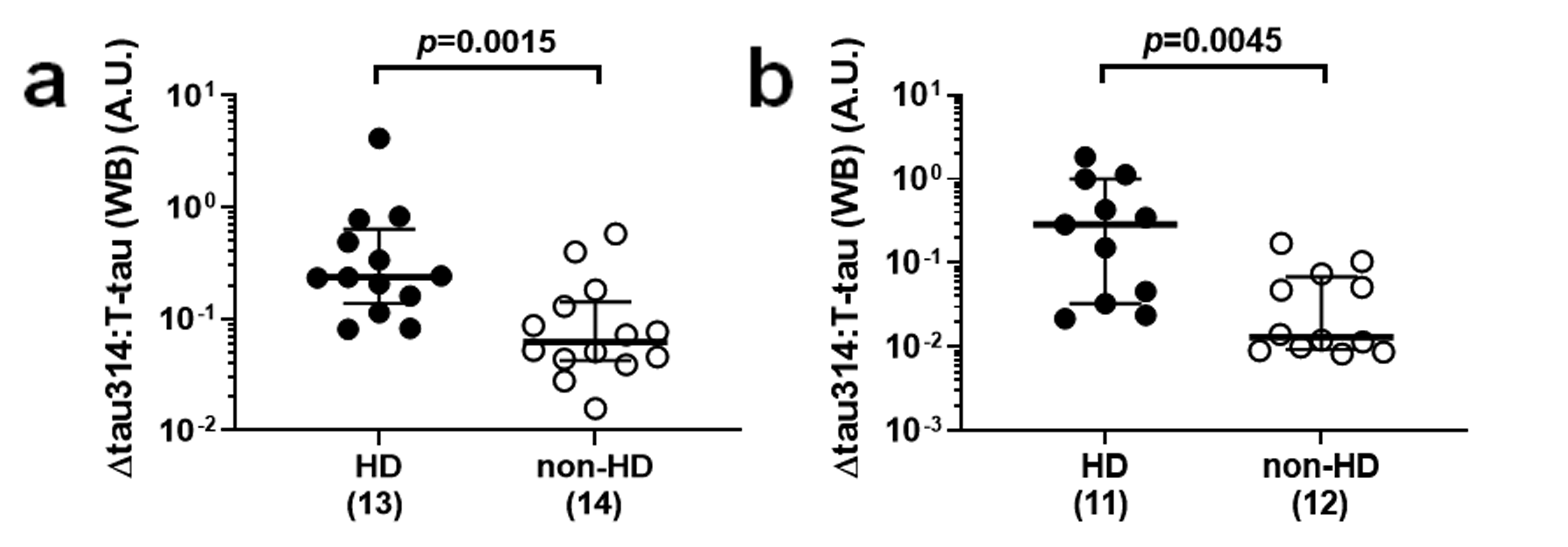

Supplement: Supplementary file 1 — Figure S1. Levels of tau-5-reactive soluble proteins revealed by direct Western blotting are lowered in HD patients than non-HD individuals. Figure S2. Levels of tau-13-immunoreactive Δtau314 proteins, following normalization to levels of tau-5-reactive proteins (T-tau (WB)) revealed by direct Western blotting, are higher in HD patients than non-HD individuals. Figure S3. Levels of glyceraldehyde 3-phosphate dehydrogenase (GAPDH) are comparable between HD patients and non-HD individuals of the large cohort. Figure S4. The relationships of Δtau314 protein levels with demographic characteristics of subjects. Table S1. Demographic and neuropathological characteristics of human subjects. Table S2. Comparison of demographic characteristics of HD patients and non-HD individuals from the HUB-ICO-IDIBELL Biobank, Spain used in the study of proteins in the prefrontal cortex (BA8). Table S3. A statistical comparison of protein levels of HD patients and non-HD individuals from the HUB-ICO-IDIBELL Biobank, Spain. Table S4. A statistical comparison of levels of proteins revealed by direct Western blotting (WB) probed with tau-5 antibody in the prefrontal cortex (BA8/9) of HD patients and non-HD individuals from the NIH NeuroBioBank and the New York Brain Bank. Table S5. A statistical comparison of protein levels of proteins revealed by direct Western blotting (WB) probed with tau-5 antibody in the caudate nucleus of HD patients and non-HD individuals from the NIH NeuroBioBank. File S1 Supplementary references. (ZIP 8370 kb) [file 40478_2019_764_MOESM1_ESM.zip › Figure S2.tif]

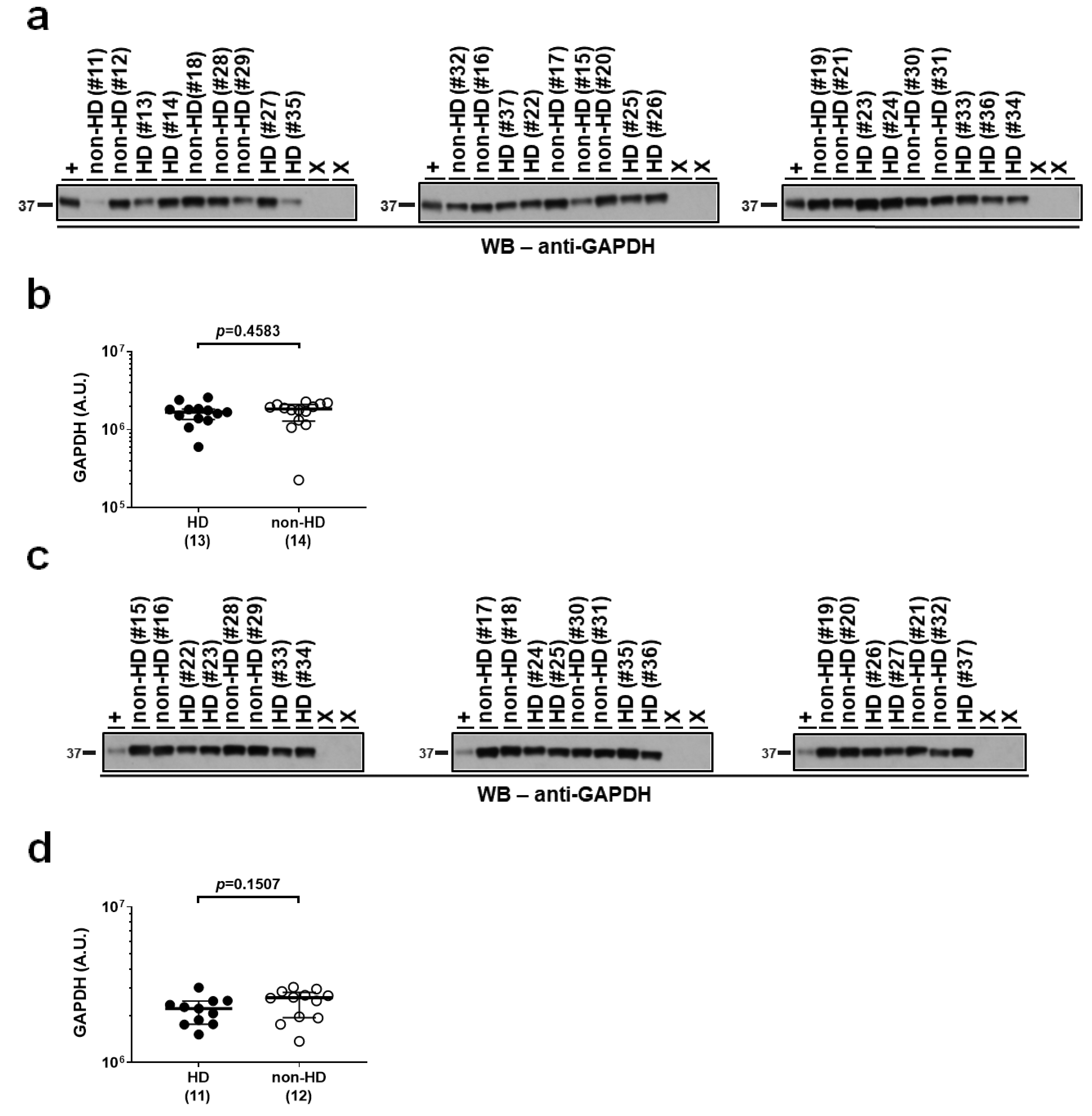

Supplement: Supplementary file 1 — Figure S1. Levels of tau-5-reactive soluble proteins revealed by direct Western blotting are lowered in HD patients than non-HD individuals. Figure S2. Levels of tau-13-immunoreactive Δtau314 proteins, following normalization to levels of tau-5-reactive proteins (T-tau (WB)) revealed by direct Western blotting, are higher in HD patients than non-HD individuals. Figure S3. Levels of glyceraldehyde 3-phosphate dehydrogenase (GAPDH) are comparable between HD patients and non-HD individuals of the large cohort. Figure S4. The relationships of Δtau314 protein levels with demographic characteristics of subjects. Table S1. Demographic and neuropathological characteristics of human subjects. Table S2. Comparison of demographic characteristics of HD patients and non-HD individuals from the HUB-ICO-IDIBELL Biobank, Spain used in the study of proteins in the prefrontal cortex (BA8). Table S3. A statistical comparison of protein levels of HD patients and non-HD individuals from the HUB-ICO-IDIBELL Biobank, Spain. Table S4. A statistical comparison of levels of proteins revealed by direct Western blotting (WB) probed with tau-5 antibody in the prefrontal cortex (BA8/9) of HD patients and non-HD individuals from the NIH NeuroBioBank and the New York Brain Bank. Table S5. A statistical comparison of protein levels of proteins revealed by direct Western blotting (WB) probed with tau-5 antibody in the caudate nucleus of HD patients and non-HD individuals from the NIH NeuroBioBank. File S1 Supplementary references. (ZIP 8370 kb) [file 40478_2019_764_MOESM1_ESM.zip › Figure S3.tif]

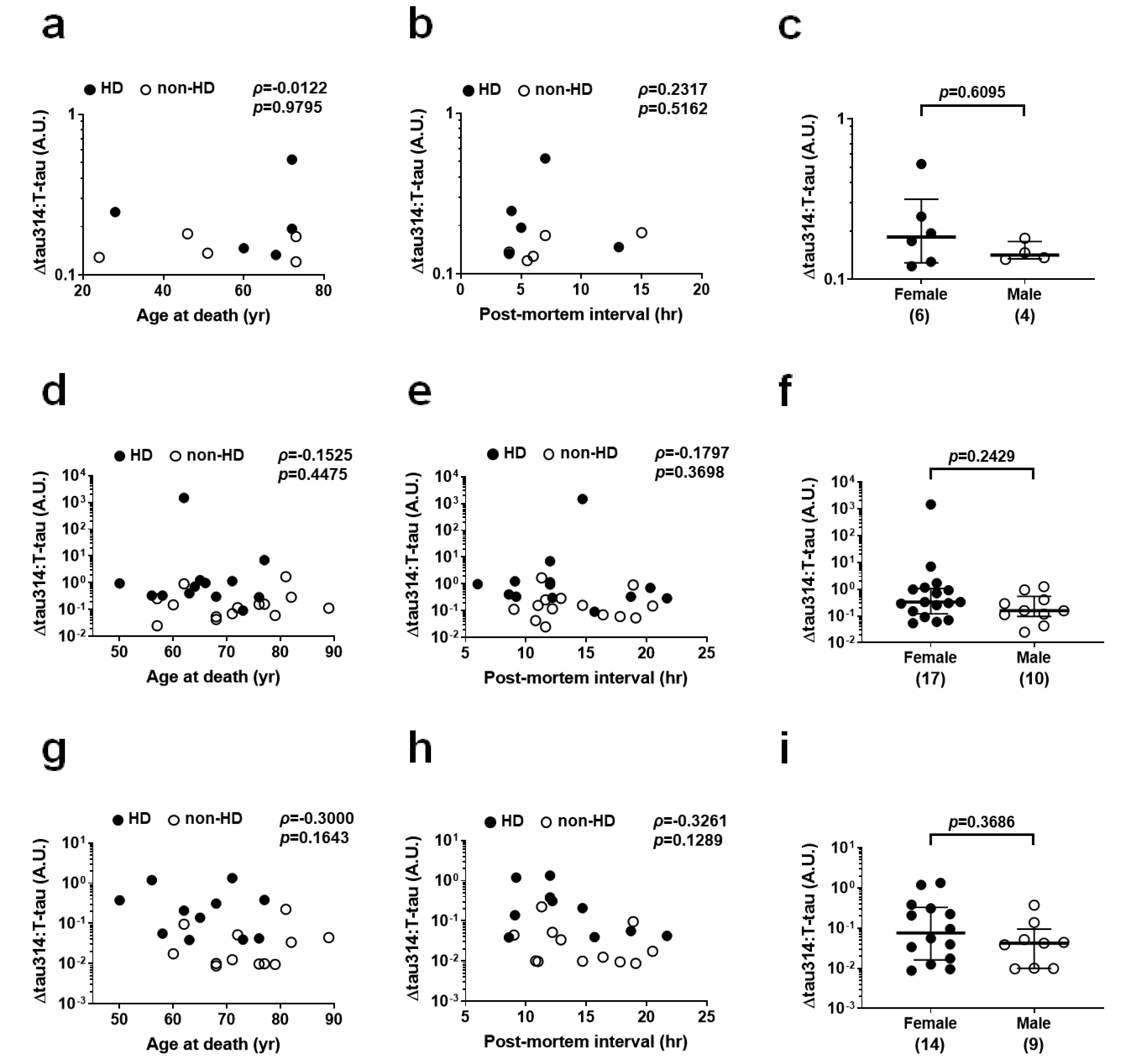

Supplement: Supplementary file 1 — Figure S1. Levels of tau-5-reactive soluble proteins revealed by direct Western blotting are lowered in HD patients than non-HD individuals. Figure S2. Levels of tau-13-immunoreactive Δtau314 proteins, following normalization to levels of tau-5-reactive proteins (T-tau (WB)) revealed by direct Western blotting, are higher in HD patients than non-HD individuals. Figure S3. Levels of glyceraldehyde 3-phosphate dehydrogenase (GAPDH) are comparable between HD patients and non-HD individuals of the large cohort. Figure S4. The relationships of Δtau314 protein levels with demographic characteristics of subjects. Table S1. Demographic and neuropathological characteristics of human subjects. Table S2. Comparison of demographic characteristics of HD patients and non-HD individuals from the HUB-ICO-IDIBELL Biobank, Spain used in the study of proteins in the prefrontal cortex (BA8). Table S3. A statistical comparison of protein levels of HD patients and non-HD individuals from the HUB-ICO-IDIBELL Biobank, Spain. Table S4. A statistical comparison of levels of proteins revealed by direct Western blotting (WB) probed with tau-5 antibody in the prefrontal cortex (BA8/9) of HD patients and non-HD individuals from the NIH NeuroBioBank and the New York Brain Bank. Table S5. A statistical comparison of protein levels of proteins revealed by direct Western blotting (WB) probed with tau-5 antibody in the caudate nucleus of HD patients and non-HD individuals from the NIH NeuroBioBank. File S1 Supplementary references. (ZIP 8370 kb) [file 40478_2019_764_MOESM1_ESM.zip › Figure S4.tif]
